# Risk of Acquired Cholesteatoma and External Auditory Canal Stenosis in Traumatic Brain Injury: A Nationwide Population-Based Cohort Study

**DOI:** 10.3390/ijerph17186624

**Published:** 2020-09-11

**Authors:** Hung-Che Lin, Cheng-Ping Shih, Hsin-Chien Chen, Chun-An Cheng, Yuahn-Sieh Huang, Chen-Shien Lin, Chi-Hsian Chung, Bor-Rong Huang, Jih-Chin Lee, Wei-Chuan Shangkuan, Wu-Chien Chien, Chi-Ming Chu

**Affiliations:** 1 Graduate Institute of Medical Sciences, National Defense Medical Center, Taipei 11490, Taiwan; lhj50702@gmail.com (H.-C.L.); zhengping_shi@yahoo.com.tw (C.-P.S.); 2 Department of Otolaryngology-Head and Neck Surgery, Tri-Service General Hospital, National Defense Medical Center, Taipei 11490, Taiwan; acolufreia@yahoo.com.tw (H.-C.C.); hbr3705@gmail.com (B.-R.H.); doc30450@gmail.com (J.-C.L.); 3 Hualien Armed Forces General Hospital, Hualien County 97144, Taiwan; 4 Department of Neurology, Tri-Service General Hospital, National Defense Medical Center, Taipei 11490, Taiwan; cca@mail.ndmctsgh.edu.tw; 5 Department of Biology and Anatomy, National Defense Medical Center, Taipei 11490, Taiwan; anatoman2001@yahoo.com.tw; 6 Chinese Medicine Department, Tri-Service General Hospital, National Defense Medical Center, Taipei 11490, Taiwan; chenshien.lin@gmail.com; 7 School of Public Health, National Defense Medical Center, Taipei 11490, Taiwan; g694810042@gmail.com; 8 Department of Medical Research, Tri-Service General Hospital, National Defense Medical Center, Taipei 11490, Taiwan; 9 Department of Otolaryngology-Head and Neck Surgery, Taipei City Hospital, Taipei City Government, Taipei 11146, Taiwan; melonkuan@gmail.com; 10 Graduate Institute of Life Sciences, National Defense Medical Center, 9314R, No.161, Section 6, Min-Chuan East Road, Neihu District, Taipei City 11490, Taiwan; 11 Taiwanese Injury Prevention and Safety Promotion Association, 4112R, No.161, Section 6, Min-Chuan East Road, Neihu District, Taipei City 11490, Taiwan

**Keywords:** traumatic brain injury, acquired cholesteatoma, external auditory canal stenosis, National Health Insurance Research Database (NHIRD)

## Abstract

The aim of study is to investigate the risk of developing acquired cholesteatoma and external auditory canal (EAC) stenosis after traumatic brain injury (TBI) from the Taiwan National Health Insurance Research Database (NHIRD). Each subject was individually traced from their index date to identify those who received a diagnosis of acquired cholesteatoma and EAC stenosis. Cox regression analyses were applied to determine the risk of TBI-related acquired cholesteatoma and EAC stenosis. The follow-up data collected over 10 years were obtained from the TBI and comparison cohorts, of 455,834 and 911,668 patients, respectively. Multivariate analysis demonstrated that TBI significantly increased the risk of cholesteatoma (adjusted hazard ratio (HR), 1.777; 95% confidence interval (CI), 1.494−2.114, *p* < 0.001) and EAC stenosis (adjusted (HR), 3.549; 95% (CI), 2.713−4.644, *p* < 0.001). In our subgroup injury analysis, falls had the highest associated risk (4.308 times), followed by traffic injuries (66.73%; 3.718 times that of the control group). Otolaryngologists should not neglect the clinical importance and carefully investigate the possibility of subsequent cholesteatoma and EAC stenosis, which leads to hearing impairment in patients with TBI. Our research also shows the important role in preventing TBI, especially as a result of traffic injuries and falls.

## 1. Introduction

Traumatic brain injury (TBI) may lead to temporal bone fracture. In 1994, Kelley and Tami classified temporal bone fractures as otic capsule-sparing or otic capsule-disrupting rather than longitudinal or transverse fractures due to the better applicability of this classification for both prognosis prediction and treatment [1]. Dahiya et al. reported that only 5.6% of patients with head injury had otic capsule-disrupting fractures. Compared with otic capsule-sparing fractures, otic capsule-disrupting fractures had seven times the probability of causing profound hearing loss [2].

Acquired cholesteatoma and external auditory canal (EAC) stenosis may result from temporal bone fracture and cause hearing loss. Hearing loss causes a significant inconvenience in daily life. However, few studies have reported these phenomena, although it is of clinical importance to be aware of the possible mechanism of post-traumatic acquired cholesteatoma and EAC stenosis related to TBI. The typical location associated with temporal bone fracture is the epitympanic region and antrum. Posttraumatic canal cholesteatoma can be prevented by close clinical observation, adequate wound care, and canal stenting for canal stenosis. The auditory canal can be dilated with several types of instruments to prevention the maturation of stenosis, which is relatively difficult to resolve. The incidence of acquired EAC stenosis is 0.6 cases per 100,000 inhabitants [3].

To date, epidemiological and clinical studies in the Asian population are lacking. In 1984, Brookes and Graham reported post-traumatic cholesteatoma and stenosis of the external auditory canal. They reported that this phenomenon is a rare condition which is not mentioned in any of the United States or United Kingdom otological literature [4] Thus, we conducted a nationwide population-based cohort study to determine the risk of acquired cholesteatoma and EAC stenosis in patients with TBI in Taiwan.

## 2. Materials and Methods

### 2.1. Data Sources

The National Health Research Institute (NHRI) of Taiwan has maintained a large computerized administrative database assembled from the National Health Insurance (NHI) medical records, including data on outpatient visits, hospital admissions, prescriptions, and disease status for all insurant. The NHI program covers 99.9% of the 23.74 million people residing in Taiwan [5,6]. The NHRI encrypts patients’ personal information for privacy protection and provides researchers with anonymous identification numbers associated with relevant claim information, including patient sex, date of birth, registry of medical services, and medication prescriptions. Patient consent is not required for accessing the NHIRD. The diagnoses and procedures are coded in the International Classification of Disease, Ninth Revision, Clinical Modification (ICD−9-CM) format. The study was approved by the Ethics Institutional Review Board of the Tri-Service General Hospital (TSGHIRB No. 2-105-05-025).

### 2.2. Variable Definitions

In our research, brain injuries in inpatients included skull fracture (ICD-9-CM 800-804) and intracranial injury (ICD-9-CM 850-854). The event of tracking definite as inpatient with acquired stenosis of the external ear canal (ICD-9-CM 380.5) and cholesteatoma of the middle ear and mastoid (ICD-9-CM 385.3). To reduce bias, we excluded patients who had the brain injury before 2000, with events before follow-up and with an unknown gender. Lengths of days represents the number of days in the hospital. All study participants were followed from the index date until the onset of acquired cholesteatoma and EAC stenosis.

### 2.3. Study Sample Selection and Tracking

**Figure 1:**
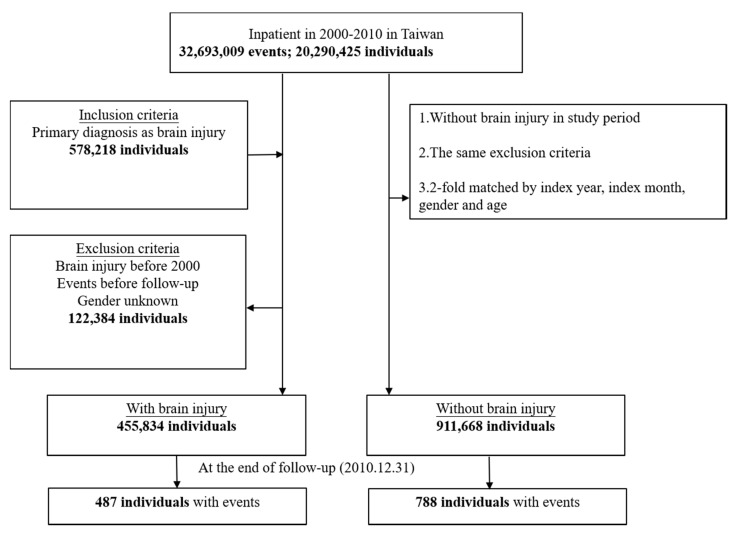
Flowchart of study sample selection from the National Health Insurance Research Database in Taiwan.

Among the 20,290,425 individuals recorded in the inpatient database from 1 January 2000 to 31 December 2010, 578,218 individuals were inpatients due to a brain injury. We excluded inpatients with brain injuries from 1997 to 1999, those with events (acquired cholesteatoma and EAC stenosis) occurring before follow-up, and those with an unknown gender; finally, 455,834 individuals were included in the case group. The control group was based on the same exclusion criteria as the study group but excluded cases of brain injury during the study period. As a result, the control group excluded the skull fracture (ICD-9-CM 800-804) and the intracranial injury (ICD-9-CM 850-854). Twice as many individuals were included in the control group, matched by index year, index month, gender and age; that is, the control group included 911,668 individuals. The tracking of the subjects continued until 31 December 2010 (Figure 1, flowchart).

### 2.4. Statistical Analysis

Continuous variables are presented as the means ± SD, and categorical variables are presented as frequencies and percentages; moreover, we used the chi-square/Fisher’s exact test and the *t*-test to compare the difference between patients with and without brain injury at the baseline and at the endpoint. Kaplan–Meier curves and Cox’s proportional hazard regression analysis were performed to calculate crude and adjusted hazard ratios (HRs), with 95% confidence intervals (CIs), to assess the effect of the brain injuries. To investigate the interaction between the demographic variables, we also calculated adjusted HRs stratified by gender, low-income households, catastrophic illness, intentionality of injury, inpatient season, urbanization level, level of care, and surgery. All statistical analyses were performed with SPSS Statistics, Version 22.0. Armonk, NY: IBM Corp., and a two-tailed *p* < 0.05 was considered as the threshold for significant variance.

### 2.5. Data Availability

The NHIRD data are available from the National Health Insurance Administration, Ministry of Health and Welfare in Taiwan for researchers who meet the criteria for access to confidential data. (Available online: https://nhird.nhri.org.tw/ (accessed on 10 September 2020)).

## 3. Results

The TBI and comparison cohorts included 455,824 and 911,668 patients, respectively. Table 1 shows the sociodemographic variables and causes of injury for the acquired cholesteatoma and EAC stenosis and control patients. No significant differences in age distribution were found between the TBI and comparison cohorts (mean age 42.90 ± 22.18 y vs 45.17 ± 25.71 y, respectively). Additionally, the majority of the patients in the brain injury cohort were male (59.32%). Over 66% of the injuries in our study causing acquired cholesteatoma and EAC stenosis in the TBI group were the result of traffic injuries. Falls were found to be the second most common mechanism of injury in the TBI group, followed by homicide and crushing. In the non-TBI group, the most common cause of injury was traffic injuries, followed by falls and other unintentional injuries. Participants in both cohorts had monthly income levels that were not considered low-income and tended to live in more urbanized areas (66.78% vs 76.42% for high and middle urbanization levels, respectively). At the end of the follow-up, the TBI group had a higher risk of developing acquired cholesteatoma and EAC stenosis than the non-TBI control group (*p* < 0.001) (Table 1 and Table S1).

**Figure 2:**
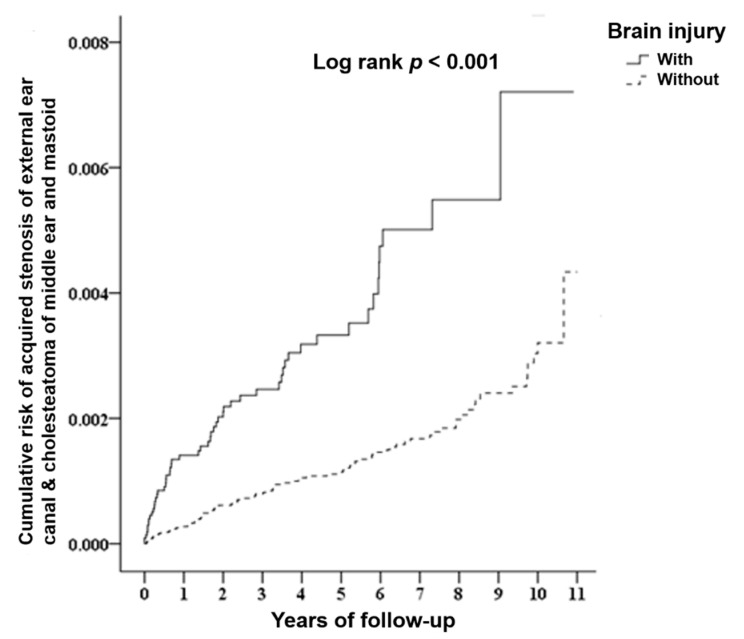
Kaplan–Meier curve of the cumulative risk of events (acquired stenosis of the external ear canal and cholesteatoma of the middle ear and mastoid) stratified by brain injury with the log-rank test.

Table 2 reveals the Cox regression analysis results of the risk of developing acquired cholesteatoma and EAC stenosis in the TBI group compared with the non-TBI control group. The adjusted HR was 2.742 (95% CI: 2.380−3.160, *p* < 0.001). The results revealed that patients with brain injury had a 2.742-fold (95% CI: 2.380−3.160) higher risk of developing acquired cholesteatoma and EAC stenosis compared with individuals without brain injury (Table 2). Kaplan–Meier analysis showed that, compared with the matched controls, patients with brain injury had a significantly higher incidence of acquired cholesteatoma and EAC stenosis (log-rank test *p* < 0.001) (Figure 2).

Table 3 shows the stratification analysis of the cause of injury associated with the risk of acquired cholesteatoma and EAC stenosis in the TBI group compared with the control group after controlling for other factors. The TBI group was associated with a higher risk of acquired cholesteatoma and EAC stenosis compared with the control group. The subgroup of traffic injuries, the risk of acquired cholesteatoma and EAC stenosis in the TBI group was 3.718-fold greater than that in the non-TBI subgroup (*p* < 0.001). In the Fall subgroup of the TBI group, the risk of acquired cholesteatoma and EAC stenosis was 4.308-fold greater than that in the non-TBI subgroup (*p* < 0.001). Our data showed that over the 10-year follow-up, 487 brain injury patients developed acquired cholesteatoma and EAC stenosis with an overall rate of 72.58 cases per 100,000 person-years, whereas 788 non-brain injury individuals had acquired cholesteatoma and EAC stenosis with an overall rate of 37.71 cases per 100,000 person-years (Table 4).

Table 4. shows the distribution of the subgroup of events in patients with acquired cholesteatoma and EAC stenosis at the end of follow-up using Cox regression. Acquired cholesteatoma accounted for the highest percentage (62.4%), followed by acquired EAC stenosis (34.1%) and both (3.5%) (cholesteatoma of the middle ear and mastoid, acquired stenosis of the external ear canal). The highest adjusted HR was for both, which was 4.099-fold greater than that for the control group, followed by acquired stenosis of the external ear canal (3.549) and cholesteatoma of the middle ear and mastoid (1.777). The average follow-up in both groups was approximately 6.1 years.

## 4. Discussion

To our knowledge, this is the first large-scale nationwide population cohort study investigating the association between TBIs, acquired cholesteatoma, and EAC stenosis. Based on a subgroup analysis, we also determined the causes of TBI that resulted in acquired cholesteatoma and EAC stenosis, such as traffic injuries and falls.

We found that patients with TBI had an increased risk of acquired cholesteatoma and EAC stenosis (adjusted HR = 2.472). Males also had a higher risk of acquired cholesteatoma and EAC stenosis than females (adjusted HR = 1.152). This result may be due to differences in daily activities and work circumstances between males and females. Males are more prone to TBI than females, and 59.32% of the patients in our study were males. Mary et al. conducted a study including patients with TBI in 46 countries, and over 81% patients were males [7]. Furthermore, Louw reported that cholesteatoma had a male predominance [8].

Jamshid proposed that most of the deaths from TBI occurring in the first week are from intracranial hypertension, and the results may support our explanations, such that the percentage of acquired cholesteatoma and EAC stenosis may be underestimated [9]. After TBI occurred, the *p* values in the brain injury group were statistically significant from the beginning of TBI to 11 years, i.e., brain injuries are truly related to acquired cholesteatoma and EAC stenosis.

In Taiwan, most of the medical centers are located in highly urbanized areas. High urbanization-level hospitals receive more TBI patients and those with more complicated disease. This may explain why the incidence of acquired cholesteatoma and EAC stenosis is greater in more urbanized areas. Furthermore, patients in medical centers had a higher risk of acquired cholesteatoma and EAC stenosis after follow-up (adjusted HR = 7.254). This may be explained by the fact that medical centers receive patients with more severe and complicated disease, so the risk of acquired cholesteatoma and EAC stenosis was the highest. Patients in medical centers often have several morbidities and a more complicated illness status.

Furthermore, surgery was associated with a higher risk of acquired cholesteatoma and EAC stenosis (adjusted HR = 7.408), which may be the reason why patients who need surgical intervention have a higher severity of TBI damage and are more prone to acquired cholesteatoma and EAC stenosis. The indications of surgical evacuation to remove space-occupying lesions such as intracranial hematomas are not only well accepted but also have favorable outcomes [10]. Patients with TBI who received surgical intervention probably had a relatively better survival, making long-term follow-up more feasible. Therefore, it may also be another reason why acquired cholesteatoma and EAC stenosis had the highest HRs in patients who underwent surgery.

The subgroup of events analysis revealed that the highest adjusted HR (4.099) was for patients with both acquired stenosis of EAC and cholesteatoma compared with acquired stenosis of EAC (3.549) and cholesteatoma (1.777). Although the incidence was only 3%, these cases were the most difficult due to the complicated otology condition. We cannot ignore such serious otology complications after TBI, so our study provides hints to Otolaryngologists to be aware of such difficulties.

The mechanisms of acquired cholesteatoma related to brain injury were as follows:

The first cause was epithelial entrapment at the fracture line located in the epitympanum and antrum, causing cholesteatoma formation. The second cause was the ingrowth of epithelium through the fracture line. The third cause was implantation of tympanic membrane skin into the middle ear by a traumatic mechanism, which led to cholesteatoma formation and a chronic inflammatory cascade that may result in medial canal fibrosis [11]. Louw proposed that trauma may play an important role in cholesteatoma pathogenesis [7]. The fourth mechanism is epithelium trapped in the EAC, leading to canal cholesteatoma. This can be prevented by close follow up using debridement and stenting. If early prevention of stenosis of the EAC is not successful, hearing impairment will occur and surgical interventions, such as canalplasty, will be required. The most common sites of traumatic cholesteatoma are the attic, antrum, and mastoid air cells. However, it often grows gradually for a while before it is detected. If cholesteatoma erodes the ossicular chain, Otolaryngologists would find conductive hearing loss. If erosion of the labyrinth occurs, symptoms of vertigo or sensorineural hearing loss would be present. If erosion of the facial nerve occurs, facial paresis would occur. However, few studies have published results on the acquired post-inflammatory stenosis of the auditory canal, not only the etiology but also the progression. Chronic inflammation will replace the original EAC epithelium by fibrotic tissue, which leads to complete occlusion of the EAC. Patients with acquired stenosis of the EAC will have severe conductive hearing loss [12]. McKennan and Chole reported that trauma may lead to EAC stenosis. Furthermore, delayed or complete closure may occur after several years. However, it is often overlooked by Otolaryngologists. It also related to canal cholesteatoma [13]. Becker and Tos proposed that the posttraumatic membranous atresia of the EAC may result from gunshots, motor vehicle accidents, and previous surgery [10]. Magliulo reported that the main cause of acquired stenosis of the EAC was chronic infection (54.1%), followed by postsurgical (20.2%) and trauma (11%) [14].

Lozano et al. proposed that TBI is related to a series of cascades, including excitotoxicity, oxidative stress, mitochondrial dysfunction, blood–brain barrier disruption, and inflammation. In the acute stage, neuroinflammation mobilizes immune cells, astrocytes, cytokines, and chemokines toward the site of injury to mount an anti-inflammatory response against brain damage. In contrast, the excess activation of these inflammatory elements in the chronic stage contributes to the secondary cell death in TBI [15].

It is well known that the pathogenesis of acquired cholesteatoma is related to the cascade of molecular events and inflammatory mediators [16]. Furthermore, TBI may result in hypoxia. Louw reported that hypoxia stimulates the hypoxia inducible factor (HIF) and increases the production of matrix metalloproteinase (MMP). It may lead to not only damage of the tympanic membrane but also bone erosion [16].

Maniu et al. reported that MMP had a vital role as a proteolytis enzyme that correlated with the aggressiveness of cholesteatoma and bony destruction [17]. Lipid peroxidation with the production of hydroperoxide and aldehydes will cause tympanic membrane deterioration. Tumor necrosis factor-α (TNF-α) and prostaglandin E2 (PGE2) were stimulated by lipopolysaccharides (LPSs). LPSs were also correlated with nitric oxide synthase activity and nitric oxide production, which contribute to bone erosion [15]. Another important growth factor is NF-κB, which is related to inflammatory and infection processes. It may be activated by TNF-α and PGE2. Jayakumar et al. proposed that NF-κB is also activated by trauma and that the activation of astrocytic NF-κB plays an important role in TBI-related cytotoxic brain edema [17]. Hamajima et al. reported that NF-κB activates the expression of cyclin D1, leading to enhancement of the progression of keratinocytes in the cell cycle [16,18,19].

The long-term follow-up up of the cumulative risk of acquired cholesteatoma and EAC stenosis by the Kaplan–Meier method revealed statistical significance, with a log-rank *p* < 0.001. Patients with TBI who had acquired cholesteatoma and EAC stenosis developed these pathologies not only shortly after trauma but also over time in our study.

In our injury subgroup analysis, the majority of TBI patients were associated with traffic injuries (66.73%), which had the second highest risk of acquired cholesteatoma and EAC stenosis, which was 3.718 times that of the control group. The second highest risk was falls, which occurred at an incidence 4.308 times that of the control group.

Thus, traffic injuries and falls are highly related to acquired cholesteatoma and EAC stenosis, such that we cannot neglect the impact of trauma on acquired cholesteatoma and EAC stenosis. The clinical implication of this finding is that otology examination, such as otoscope examination or temporal bone CT, may need to be performed early after brain injury to identify acquired cholesteatoma and EAC stenosis, enabling treatment of the cholesteatoma and EAC stenosis as early as possible to prevent hearing loss. Prevention of pathology is the best policy, and our data show that our government may need to take some measures to prevent traffic injuries and falls from occurring. Adequate prevention of TBI may not only reduce the number of patients with acquired cholesteatoma and EAC stenosis but may also reduce health care costs.

The strength of this study lies primarily in the large sample size obtained from our nationwide database. No previous large-scale studies have sought to link TBI to acquired cholesteatoma and EAC stenosis. One reason may be due to the small sample size typical of individual institutions. Another reason may be the difficulty of performing long-term follow-up. The greatest clinical value of our study was the 10-year follow-up. Our nationwide study provides strong evidence of the correlation between TBI, cholesteatoma, and EAC stenosis.

However, this study has several limitations. First, no detailed clinical information was obtained, including Glasgow Coma Scale scores, clinical manifestations on computed tomography of the head, pure tone audiogram (PTA) data, or other otology exams. Furthermore, metaplasia theory is one of theory of pathogenesis in acquired cholesteatoma and correlated with chronic or recurrent otitis media. The columnar epithelium will shift to keratinized stratified squamous epithelium. However, Vennix et al. conduct a study and showed that metaplasia is not thought to be a significant cause of cholesteatoma [20]. As a result, the impact of lacking in detail otology exam information in NHIRD, such as clinical pictures in patients with eardrum perforation, is relatively reduced. Second, the NHIRD documents only the date of cholesteatoma and EAC stenosis acquisition but does not include the severity. The effect of brain injury on the severity of acquiring cholesteatoma and EAC stenosis cannot be analyzed. Third, both ICD ninth and tenth revisions do not contain a detailed type of temporal bone fracture code and all temporal bone fractures were classified into skull base fractures. Further detailed classification in a new ICD revision edition for temporal bone fracture is of paramount importance in clinical practice. That is, it would be more meaningful if it could describe the type of skull fractures among these patients. Fourth, the tables did not include medication other than TBI. Thus, further investigation of medication efficacy in patients with acquired cholesteatoma and EAC Stenosis in TBI is needed. Lastly, during this ten-year follow-up period, the timing of the event is not consistent so that the control group pairing also varies with the time of each event. That is, not every event happens on 1 January 2000. Therefore, the tracking time for each event is not all ten years. The average follow-up in both groups was approximately 6.1 years rather than 10 years.

## 5. Conclusions

This study demonstrated an association between TBI and the subsequent development of acquired cholesteatoma and EAC stenosis by a long-term follow-up. We suggest that Otolaryngologists cannot neglect the clinical importance and carefully investigate the possibility of subsequent cholesteatoma and EAC stenosis, which leads to hearing impairment in patients with TBI. Our research also shows the important role of preventing TBI from occurring, especially in cases due to traffic injuries and falls.

## Figures and Tables

**Table 1:** Characteristics of study in the end of follow-up.

Brain Injury	Total		With		Without		*p*
Variables	*n*	%	*n*	%	*n*	%	
Total	1367,502		455,834	33.33	911,668	66.67	
Events							<0.001
Without	1366,227	99.91	455,347	99.89	910,880	99.91	
With	1275	0.09	487	0.11	788	0.09	
Subgroup of events							<0.001
Without	1366,227	99.91	455,347	99.89	910,880	99.91	
Acquired stenosis of external ear canal	355	0.03	166	0.04	189	0.02	
Cholesteatoma of middle ear and mastoid	883	0.06	304	0.07	579	0.06	
Both	37	0.00	17	0.00	20	0.00	
Gender							0.999
Male	811,257	59.32	270,419	59.32	540,838	59.32	
Female	556,245	40.68	185,415	40.68	370,830	40.68	
Age (years) (mean ± SD)	44.41 ± 24.61		42.90 ± 22.18		45.17 ± 25.71		0.007
Low-income							<0.001
Without	1344,640	98.33	447,731	98.22	896,909	98.38	
With	22,862	1.67	8103	1.78	14,759	1.62	
Catastrophic illness							<0.001
Without	1193,522	87.45	414,727	91.52	778,795	85.43	
With	171,280	12.55	38,407	8.48	132,873	14.57	
CCI (mean ± SD)	0.84 ± 2.25		0.42 ± 1.43		1.05 ± 2.53		<0.001
Cause of injury							<0.001
Traffic injuries	282,937	61.50	233,142	66.73	49,795	44.99	
Poisoning	1833	0.40	401	0.11	1432	1.29	
Falls	95,781	20.82	74,447	21.31	21,334	19.27	
Burns and fires	519	0.11	40	0.01	479	0.43	
Drowning	122	0.03	24	0.01	98	0.09	
Suffocation	636	0.14	24	0.01	612	0.55	
Crushing/Cutting/Piercing	27,002	5.87	13,773	3.94	13,229	11.95	
Other unintentional injuries	22,822	4.96	5963	1.71	16,859	15.23	
Suicide	2055	0.45	248	0.07	1807	1.63	
Homicide/Abuse	25,162	5.47	20,872	5.97	4290	3.88	
Intention unknown	1174	0.26	422	0.12	752	0.68	
Intentionality of injury							<0.001
Unintentional injury	382,674	93.89	295,298	93.64	87,376	94.76	
Intentional injury	0.00	6.11	20,050	6.36	4835	5.24	
Urbanization level							<0.001
High	414,921	30.34	114,667	25.16	300,254	32.93	
Middle	585,726	42.83	189,281	41.52	396,445	43.49	
Low	366,855	26.83	151,886	33.32	214,969	23.58	
Level of care							<0.001
Hospital center	419,434	30.67	111,842	24.54	307,592	33.74	
Regional hospital	555,404	40.61	201,180	44.13	354,224	38.85	
Local hospital	392,664	28.71	142,812	31.33	249,852	27.41	
Surgery							<0.001
Without	854,414	62.48	313,931	68.87	540,483	59.29	
With	513,088	37.52	141,903	31.13	371,185	40.71	
Length of days (mean ± SD)	7.32 ± 9.60		7.54 ± 10.13		6.89 ± 8.41		<0.001
Medical costs (NT$) (mean ± SD)	46,252.58 ± 84,924.68		48,896.69 ± 90,655.82		40,964.37 ± 71,818.28		<0.001
Prognosis							<0.001
Survive	1281,971	93.75	440,530	96.64	841,441	92.30	
Mortality	85,531	6.25	15,304	3.36	70,227	7.70	

**Table 2:** Factors of acquired stenosis of external ear canal and cholesteatoma of middle ear and mastoid in the end of follow-up by using Cox regression.

Variables	Crude HR	95% CI	95% CI	*p*	Adjusted HR	95% CI	95% CI	*p*
Brain injury								
Without	Reference				Reference			
With	2.827	2.518	3.174	<0.001	2.742	2.380	3.160	<0.001
Gender								
Male	1.121	1.003	1.253	0.044	1.152	1.029	1.290	0.014
Female	Reference				Reference			
Age (years)	0.984	0.882	1.007	0.723	0.993	0.890	1.015	0.160
Low-income								
Without	Reference				Reference			
With	0.848	0.579	1.241	0.396	1.334	0.910	1.957	0.140
Catastrophic illness								
Without	Reference				Reference			
With	0.328	0.263	0.410	<0.001	0.964	0.755	1.232	0.772
CCI	0.493	0.449	0.542	<0.001	0.702	0.642	0.767	<0.001
Intentionality of injury								
Unintentional injury	Reference				Reference			
Intentional injury	0.814	0.539	1.229	0.327	0.779	0.516	1.176	0.234
Urbanization level								
High	3.318	2.760	3.989	<0.001	1.660	1.347	2.045	<0.001
Middle	2.657	2.219	3.181	<0.001	1.706	1.408	2.066	<0.001
Low	Reference				Reference			
Level of care								
Hospital center	8.280	6.487	10.568	<0.001	7.254	5.624	9.358	<0.001
Regional hospital	3.994	3.114	5.123	<0.001	4.253	3.311	5.462	<0.001
Local hospital	Reference				Reference			
Surgery								
Without	Reference				Reference			
With	10.417	8.897	12.196	<0.001	7.408	6.309	8.699	<0.001
Length of days	0.962	0.954	0.971	<0.001	0.984	0.975	0.993	<0.001
Medical costs (NT$)	1.000	0.999	1.001	0.063	Medical cost had collinearity with length of days			

HR = hazard ratio, CI = confidence interval, Adjusted HR: Adjusted variables listed in the table; CCI = Charlson Comorbidity Index; Unintentional injury: ICD-9-CM E800-E949; Intentional injury: ICD-9-CM E950-E979, E990-E999.

**Table 3:** Cholesteatoma of middle ear and mastoid in the end of follow-up stratified by variables listed in the table by using Cox regression.

Brain Injury	With			Without			Ratio	Adjusted HR	95%CI	95%CI	*p*
Variables	Event	PYs	Rate (per 10^5^ PYs)	Event	PYs	Rate (per 10^5^ PYs)					
Total	487	671,019.80	72.58	788	2089,637.43	37.71	1.925	2.742	2.380	3.160	<0.001
Gender											
Male	294	367,891.58	79.91	453	1308,410.45	34.62	2.308	2.916	2.439	3.486	<0.001
Female	193	303,128.22	63.67	335	781,226.98	42.88	1.485	2.403	1.898	3.043	<0.001
Low-income											
Without	479	653,341.38	73.32	769	2037,529.18	37.74	1.943	2.815	2.440	3.248	<0.001
With	88	17,678.44	497.78	19	52,108.25	36.46	13.652	1.764	0.279	2.092	0.601
Catastrophic illness											
Without	449	580,954.43	77.29	742	1698,403.77	43.69	1.769	2.659	2.298	3.078	<0.001
With	38	90,065.37	42.19	46	391,233.66	11.76	3.588	4.448	2.511	7.881	<0.001
Cause of injury											
Traffic injuries	269	330,660.04	81.35	45	111,516.58	40.35	2.016	3.718	2.298	4.400	<0.001
Poisoning	0	846.72	0.00	1	3465.09	28.86	0.000	0.000	-	-	0.936
Falls	59	114,645.04	51.46	10	50,033.79	19.99	2.575	4.308	2.172	8.543	<0.001
Burns and fires	0	71.50	0.00	1	972.75	102.80	0.000	0.000	-	-	0.993
Drowning	0	63.78	0.00	0	142.27	0.00	-	-	-	-	-
Suffocation	0	43.58	0.00	1	1164.33	85.89	0.000	0.000	-	-	0.994
Crushing/Cutting/Piercing	16	19,105.02	83.75	21	25,836.71	81.28	1.030	1.952	0.957	3.980	0.066
Other unintentional injuries	5	8249.28	60.61	20	42,490.77	47.07	1.288	2.119	0.776	5.791	0.143
Suicide	0	479.98	0.00	3	4477.30	67.00	0.000	0.000	-	-	0.986
Homicide / Abuse	21	35,091.05	59.84	3	7653.63	39.20	1.527	2.758	0.803	9.475	0.107
Intention unknown	1	944.27	105.90	0	1774.84	0.00	-	-	-	-	-
Intentionality of injury											
Unintentional injury	318	406,063.25	78.31	89	188,840.93	47.13	1.662	2.745	2.145	3.513	<0.001
Intentional injury	20	33,727.89	59.30	4	11,337.65	35.28	1.681	2.576	0.841	7.889	0.098
Urbanization level											
High	181	165,497.50	109.37	329	613,218.28	53.65	2.038	2.839	2.272	3.549	<0.001
Middle	229	280,303.89	81.70	393	929,405.03	42.29	1.932	2.447	1.991	3.009	<0.001
Low	77	225,218.41	34.19	69	546,951.12	12.62	2.710	3.743	2.487	5.633	<0.001
Level of care											
Hospital center	239	162,375.45	147.19	472	667,553.56	70.71	2.082	2.764	2.281	3.350	<0.001
Regional hospital	213	311,469.03	68.39	280	916,963.67	30.54	2.240	2.611	2.082	3.275	<0.001
Local hospital	35	197,175.33	17.75	36	505,120.20	7.13	2.491	3.425	1.919	6.112	<0.001
Surgery											
Without	111	402,565.91	27.57	69	1329,927.27	5.19	5.315	5.750	3.894	8.490	<0.001
With	376	268,453.89	140.06	719	759,710.16	94.64	1.480	2.388	2.047	2.789	<0.001

PYs = Person-years; Ratio = Rate in case ÷ Rate in control; Adjusted HR = Adjusted Hazard ratio: Adjusted for all the variables above; CI = confidence interval; CCI = Charlson Comorbidity Index; Unintentional injury: ICD-9-CM E800-E949; Intentional injury: ICD-9-CM E950-E979, E990-E999; Some patients did not provide the information of cause and intentionality of injury.

**Table 4:** Factors of acquired stenosis of external ear canal and cholesteatoma of middle ear and mastoid in the end of follow-up by using Cox regression.

Brain Injury	With			Without			Ratio	Adjusted HR	95%CI	95%CI	*p*
Subgroup of Events	Event	PYs	Rate (per 10^5^ PYs)	Event	PYs	Rate (per 10^5^ PYs)					
Total	487	671,019.80	72.58	788	2089,637.43	37.71	1.925	2.742	2.380	3.160	<0.001
Acquired stenosis of external ear canal	166	671,019.80	24.74	189	2089,637.43	9.04	2.735	3.549	2.713	4.644	<0.001
Cholesteatoma of middle ear and mastoid	304	671,019.80	45.30	579	2089,637.43	27.71	1.635	1.777	1.494	2.114	<0.001
Both	17	671,019.80	2.53	20	2089,637.43	0.96	2.647	4.099	1.001	21.781	0.049

PYs = Person-years; Ratio = Rate in case ÷ Rate in control; Adjusted HR = Adjusted Hazard ratio: Adjusted for variables listed in Cox sheet; CI = confidence interval.

## Supplementary Materials

The following are available online at https://www.mdpi.com/1660-4601/17/18/6624/s1, Table S1: Characteristics of study in the baseline.
