# Supplementary material for: Risk of Acquired Cholesteatoma and External Auditory Canal Stenosis in Traumatic Brain Injury: A Nationwide Population-Based Cohort Study
Source: Int J Environ Res Public Health. 2020 Sep 11;17(18):6624. doi: 10.3390/ijerph17186624 (PMC7558982; doi:10.3390/ijerph17186624)
Supplement: Supplementary file 1 [file ijerph-17-06624-s001.pdf]

Table S1. Characteristics of study in the baseline.

Brain Injury Variables	Total		With		Without		*p*
	n	%	*n*	%	*n*	%	
Total	1,367,502		455,834	33.33	911,668	66.67	
Gender							0.999
Male	811,257	59.32	270,419	59.32	540,838	59.32	
Female	556,245	40.68	185,415	40.68	370,830	40.68	
Age (years) (mean ± SD)	41.65 ± 23.75		41.45 ± 21.79		41.75 ± 24.68		0.373
Low-income							<0.001
Without	1,350,778	98.78	449,267	98.56	901,511	98.89	
With	16,724	1.22	6,567	1.44	10,157	1.11	
Catastrophic illness							<0.001
Without	1,271,995	93.02	431,689	94.70	840,306	92.17	
With	95,507	6.98	24,145	5.30	71,362	7.83	
CCI (mean ± SD)	0.48 ± 1.43		0.14 ± 0.57		0.64 ± 1.68		<0.001
Cause of injury							<0.001
Traffic injuries	282,937	61.50	233,142	66.73	49,795	44.99	
Poisoning	1,833	0.40	401	0.11	1,432	1.29	
Falls	95,781	20.82	74,447	21.31	21,334	19.27	
Burns and fires	519	0.11	40	0.01	479	0.43	
Drowning	122	0.03	24	0.01	98	0.09	
Suffocation	636	0.14	24	0.01	612	0.55	
Crushing/ Cutting/ Piercing	27,002	5.87	13,773	3.94	13,229	11.95	
Other unintentional injuries	22,822	4.96	5,963	1.71	16,859	15.23	
Suicide	2,055	0.45	248	0.07	1,807	1.63	
Homicide/Abuse	25,162	5.47	20,872	5.97	4,290	3.88	
Intention unknown	1,174	0.26	422	0.12	752	0.68	
Intentionality of injury							<0.001
Unintentional injury	382,674	93.89	295,298	93.64	87,376	94.76	
Intentional injury	24,885	6.11	20,050	6.36	4,835	5.24	

Urbanization level							<0.001
High	415,214	30.36	105,263	23.09	309,951	34.00	
Middle	575,609	42.09	186,466	40.91	389,143	42.68	
Low	376,679	27.55	164,105	36.00	212,574	23.32	
Level of care							<0.001
Hospital center	392,964	28.74	95,251	20.90	297,713	32.66	
Regional hospital	511,542	37.41	194,044	42.57	317,498	34.83	
Local hospital	462,996	33.86	166,539	36.54	296,457	32.52	
Surgery							<0.001
Without	873,691	63.89	346,649	76.05	527,042	57.81	
With	493,811	36.11	109,185	23.95	384,626	42.19	
Length of days (mean ± SD)	6.65 ± 8.85		6.83 ± 8.79		6.56 ± 8.88		<0.001
Medical costs (NT$) (mean ± SD)	35,567.90 ± 71,694.20		42,940.78 ± 91,911.65		31,811.46 ± 58,697.95		<0.001

P-value (category variable: Chi-square/Fisher exact test, continuous variable: t-test); CCI = Charlson Comorbidity Index; Traffic injuries: ICD-9-CM E800-849; Poisoning: ICD-9-CM E850-E869; Falls: ICDCM E880-E889; Burns and fires: ICD-9-CM E890-E899; Drowning: ICD-9-CM E910; Suffocation: ICD-9-CM E911-E915; Other unintentional injuries: E870-E879, E900-E909, E921-E949; Suicide: ICD-9-CM E950-E959; Homicide / Abuse: ICD-9-CM E960-E969; Intention unknown: ICD-9-CM E980-E989; Unintentional injury: ICD-9-CM E800-E949; Intentional injury: ICD-9-CM E950-E979, E990-E999. Some patients did not provide the information of cause and intentionality of injury.
